# Clinical outcomes of three-fraction versus five-fraction stereotactic radiosurgery for resected brain metastases

**DOI:** 10.1007/s11060-025-05357-7

**Published:** 2026-01-21

**Authors:** Giuseppe Minniti, Paolo Tini, Piera Navarria, Giorgio Raza, Giuseppe Maria Della Pepa, Silvia Chiesa, Francesca De Felice, Lucy Zaccaro, Luca Capone, Miriam Tomaciello, Gaetano Lanzetta, Martina Giraffa, Ivana Russo, Francesco Marampon, Antonio Bruno, Sergio Paolini

**Affiliations:** 1 Department of Radiological Sciences, Oncology, and Anatomical Pathology, Sapienza University of Rome, Policlinico Umberto I, Viale del Policlinico 155, Rome, 00161 Italy; 2 IRCCS Neuromed, Pozzilli (IS), Italy; 3 Department of Medicine, Surgery and Neurosciences, University of Siena, Siena, Italy; 4 Radiotherapy and Radiosurgery Department, IRCCS Humanitas Research Hospital, Rozzano, Milan, 20089 Italy; 5 UPMC Hillman Cancer Center, San Pietro Hospital FBF, Rome, Italy; 6 Department of Neurosurgery, Fondazione Policlinico Universitario A. Gemelli IRCCS, Largo Agostino Gemelli 8, Rome, 00168 Italy; 7 Department of Radiology, Radiotherapy and Hematology, Fondazione Policlinico Universitario A. Gemelli IRCCS, Rome, Italy; 8 UPMC Hillman Cancer Center, Villa Maria, Mirabella Eclano (AV), Italy; 9 Department of Neuroscience, Sapienza University, Rome, Italy

**Keywords:** Brain metastases, Hypofractionated stereotactic radiosurgery, Resection cavity, Radiation necrosis, Leptomeningeal disease

## Abstract

**Purpose:**

To investigate the clinical outcomes of patients with brain metastases undergoing surgery followed by postoperative three-fraction or five-fraction radiosurgery (SRS) to the resection cavity. Factors associated with local failure and symptomatic radionecrosis were evaluated.

**Patients and methods:**

One hundred and ninety consecutive patients with 202 brain metastases who received surgery followed by three- or five-fraction SRS to the surgical bed were analyzed. All cavities included in the study received either 27 Gy in 3 fractions or 30 Gy in 5 fractions given daily on consecutive days. Cumulative incidence analysis was used to compare local control and symptomatic radionecrosis between groups from the time of SRS.

**Results:**

Ten cavities after 3-fraction and 14 lesions after 5-fraction SRS group recurred (*p* = 0.38) with a median time to progression of 12 months. Cumulative LC rates were 93% (87–97) and 90.2% (87.5–94.5) at 1 year, and 90% (84–96) and 85.5% (76.2–91.3) at 2 years (*p* = 0.4) for 3-fraction and 5-fraction SRS groups, respectively. Symptomatic radionecrosis developed in 28 patients (3-fraction SRS,16; 5-fraction SRS,12), requiring surgery or medical treatment; the 1-year and 2-year cumulative rates were 12.8% (8.3–22.4%) and 17.9% (12.3–28.9%), respectively, after 3-fraction SRS, and 9.8% (6.5–19.1%) and 13.0% (7.3–22.2%), respectively, after 5-fraction SRS (*p* = 0.3). Sixteen patients after 3-fraction and fourteen patients after 5-fraction SRS developed leptomeningeal disease (LMD); 1-year LMD cumulative rates were 13.5% and 13.8%.

**Conclusions:**

Both 3 × 9 Gy and 5 × 6 Gy are effective SRS treatment modalities for resected brain metastases associated with high local control and low risk of symptomatic radionecrosis.

## Introduction

Stereotactic radiosurgery (SRS) has become the recommended treatment in patients with multiple brain metastases (BMs) [1, 2]. For resected BMs, data from randomized trials have shown that postoperative SRS to the resection cavity improves local control (LC) compared to surgery alone and reduces the risk of cognitive decline compared to the whole brain radiation therapy (WBRT) without decreasing survival [3, 4].

Using single-fraction doses of 15 to 24 Gy, randomized trials and retrospective series have shown local control rates of 70 to 90% following SRS to the surgical bed, with an estimated risk of symptomatic radionecrosis (sRN) < 10% [3–9]; however, an increased risk of local failure up to 50% has been reported for large cavities >3 cm in maximum size treated with doses < 15 Gy [3–5, 7, 8]. In addition, single-fraction SRS for large brain volumes can be associated with an increased risk of sRN [10, 11].

Hypofractionated SRS using 24–27 Gy administered in three fractions or 25–30 Gy administered in 5 fractions, has been conditionally recommended as an alternative to single-fraction SRS for large target volumes >2.5–3 cm in size to improve LC while limiting the risk of RN associated with single doses [1, 2]. Using doses of 24–35 Gy given in three to five fractions to the surgical bed, some retrospective studies showed LC rates up to 90% at 1 year, with a variable risk of sRN ranging from 2% to 25% [12–19]. Factors associated with the development of sRN include the size of the resection cavity, volume of the brain receiving specific doses, and combined systemic therapies [12, 16, 17, 20].

In the present study we have evaluated the LC and the risk of sRN in patients who received 3-fraction SRS (3 × 9 Gy) or 5-fraction SRS (5 × 6 Gy) for resected BMs. Related factors associated with the clinical outcomes and the development of sRN have been assessed.

## Patients and methods

Between March 2018 and September 2024, 232 consecutive patients aged ≥ 18 years who received complete resection of at least one BM derived from histologically confirmed systemic cancer, followed by adjuvant 3- or 5-fraction SRS using a linear accelerator (LINAC)-based system, were retrospectively evaluated for this study.

All radiographic, surgical, and pathological information was extracted from a prospectively maintained database of patients with brain tumors treated at the UPMC Hillman Cancer Centers of Rome and Mirabella Eclano (Av), and Neuromed Hospital, Pozzilli (IS), Italy. After excluding 42 patients due to ineligible treatment modalities (previous WBRT or different SRS fractionation) or insufficient clinical information, a total of 190 patients remained in the final analysis. The Institutional Review Board at San Pietro Hospital FBF approved the study.

The gross tumor volume (GTV) included the entire surgical bed, with no inclusion of surrounding areas of edema and the surgical resection corridor. To account for microscopic disease, the clinical target volume (CTV) was contoured by adding a margin of 1 mm around the resection cavity with an additional margin of at least 5 mm over the craniotomy bone flap adherent to the underlying dura for lesions presenting with preoperative dural contact. A margin of 0 (*n* = 116) or 1 mm (*n* = 86) was added around CTV to generate the planning target volume (PTV).

All resection cavities were treated with a total dose of 27 Gy administered in 3 daily fractions or 30 Gy in 5 daily fractions corresponding to a biological effective dose (BED) of 51.3 Gy and 48 Gy, respectively, using the linear quadratic (LQ) model for the estimation of dose-effect relationship, and assuming an α/β of 10 Gy for brain metastases. A similar BED of 47.25 Gy (3-fraction SRS) and 46.05 Gy (5-fraction SRS) can be observed using the linear quadratic cubic (LQC) model, which is a modified LQ model that can better estimate the effect of high doses per fraction [21].

The SRS regimen was chosen at the discretion of the radiation oncologist at each participating center. Treatments were performed with a TrueBeam LINAC system using modulated arc therapy (VMAT) technique. Doses were prescribed to the 80% isodose line to achieve a minimum 95% target coverage of the prescribed dose. Cone-beam Computed Tomography (CBCT) and the ExacTrac image-guided systems were used for setup verification before/during each fraction. For patients presenting with multiple BMs, intact lesions were treated with single-fraction SRS, 18–22 Gy, or fractionated SRS, 3 × 9 Gy.

Patients were examined clinically before treatment and subsequently every 2 months. For clinical follow-up, a detailed neurologic examination was performed, and the severity of complications was rated according to the Common Terminology Criteria for Adverse Events (CTCAE) v6.0 (https://dctd.cancer.gov/research/ctep-trials/for-sites/adverse-events/ctcae-v6.pdf**). **Symptomatic RN (sRN) was defined as imaging-confirmed radionecrosis associated with new or worsening neurological symptoms requiring medical or surgical management. Definitions of local and distant intracranial progression were based on the response assessment in neuro-oncology brain metastases (RANO-BM) working group criteria [22]. Accordingly, local progression was defined as new nodular or mass-like enhancement within the 80% isodose line, whereas distant intracranial progression included new parenchymal or leptomeningeal lesions outside the treated cavity.

The diagnosis of tumor progression versus RN was determined based on histology (for patients who underwent surgical resection) or by imaging using MRI and 3,4-dihydroxy-6-(18)F-fluoro-l-phenylalanine (F-DOPA) PET-CT [23]. Leptomeningeal disease (LMD) was defined as focal or diffuse new linear or nodular leptomeningeal enhancement of cerebral sulci, cerebellar folia, basal cisterns, spinal cord, cauda equina, and dural/pial surface (or dura) extending beyond the planning target volume [24]. Additionally, cytologic confirmation of malignant cells in the cerebrospinal fluid was also considered if appropriated.

## Data analysis

OS was estimated using the Kaplan-Meier method from the date of first SRS fraction to the date of death from any cause or censored at the date of last follow-up for survivors. Cumulative incidence function was used to estimate the probability of LC, distant brain failure (DBF), RN and LMD with death as a competing risk. Gray’s test [25] was used to test for differences in the cumulative incidence between groups. Fisher’s exact test and nonparametric Mann-Whitney tests were used to examine between-group covariate differences, and the Cox proportional hazards model was used to assess the effects of clinical/treatment variables on clinical outcomes. The proportional hazards assumption for Cox regression models was verified using Schoenfeld residuals for all covariates, with no significant violations detected (*p* >0.05). Variables at a significance level of *p* < 0.1 were included in a multivariate analysis. According to previous published risk prediction models of RN [12, 17, 19, 26], the following dose-volume constraints associated with sRN risk were evaluated: brain minus GTV receiving 20 Gy (V_20(B−GTV)_), 24 Gy (V_24(B−GTV)_), and 30 Gy (V_30(B−GTV)_), and brain plus TV receiving 20 (V_20(B+TV)_) and 24 Gy (V_24(B+TV)_). Statistical evaluation was performed using a commercial statistical software package (XLSTAT statistical software). A *p* < 0.05 was considered statistically significant.

## Results

### Patient and treatment characteristics

A total of 190 consecutive patients with 202 resected BMs were analyzed. Patient characteristics are shown in Table 1. Ninety-six patients (103 lesions) received 3-fraction and ninety-four patients (99 lesions) received 5-fraction SRS. All patients had not received previous SRS treatments before surgery. One hundred and seventy-one patients received one or two lines of systemic therapies prior to SRS. There were no statistically significant differences between groups in terms of gender, age, histology, Karnofsky performance status (KPS) scores, number of metastases, and presence of actionable driver mutations. However, patients given 3-fraction SRS were more likely to have smaller target volumes. Concurrent systemic treatments at the time of postoperative hypofractionated SRS included chemotherapy (*n* = 46), immunotherapy (*n* = 72), antibody-drug conjugates (ADCs) (*n* = 18), and targeted therapies (*n* = 36).

**Table 1:** Summary of patient characteristics and treatment parameters

Variable	Surgery plus 3-fractionSRS	Surgery plus 5-fraction SRS	*p* value
	*N* = 96	*N* = 94	
***Sex (F/M)***	52/44	50/44	NS
***Age (years)***			NS
Median	59	62	
Range	27-81	32-82	
***Histology***			NS
Breast carcinoma	11	18	
NSCLC	51	46	
Melanoma	21	20	
Colon	7	4	
Others	6	5	
**Concurrrent systemic treatment**			NS
None/CHT	32	34	
Immunotherapy	38	34	
Targeted therapy	16	20	
Antibody-Drug Conjugates	10	8	
***KPS***			
Median	90	90	NS
Range	60-100	60-100	
***Extracranial diseae***			NS
Uncontrolled	31	34	
Controlled	65	60	
***Number of cavities***			NS
Single	89	89	
Multiple	7	5	
***GTV (cm*** ^***3***^ ***)***			NS
Median	10.2	11.3	
Range	2.6 - 48.4	2.9 - 63.4	
***PTV (cm*** ^***3***^ ***)***			NS
Median	15.8	17.2	
Range	3.4 - 56.7	3.8-77.3	
Conformity index*			NS
Median	1.41	1.44	
Range	1.28-2.1	1.3-2.1	

KPS, Karnofsky Performance Status; GTV, Gross TumorVolume; PTV, Planning Target Volume

*prescribed isodose volume/tumor volume encompassed by the prescription isodose volume

All the patients received the planned dose to the resection cavity with the PTV that was covered by at least 98% of the prescription dose. The median time from surgery to the treatment was 26 days, with over 84% of patients treated within 4 weeks of surgery. In 83 patients with multiple BMs, single-fraction SRS was used for 154 lesions and hypofractionated SRS for 36 lesions. At the time of analysis (July 2025), 80 patients were still alive (3-fraction SRS, 41; 5-fraction SRS, 39).

### Brain control and survival

**Fig. 1:**
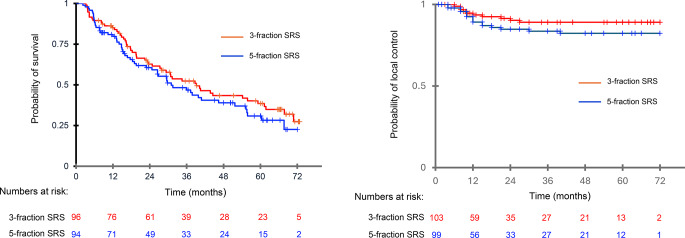
Kaplan-Meier analysis of overall survival local control after 3-fraction and 5-fraction stereotactic radiosurgery (SRS). Censored data are denoted by tick marks

After a median follow-up of 19 months, 10 cavities in 3-fraction and 14 cavities in 5-fraction SRS group recurred; median times to progression were 10 months (range, 6–27 months) and 12 months (range, 4–40 months), respectively. Cumulative LC rates were 93% (95%CI,87–98) and 90.2% (95%CI, 87.5–94.5) at 1 year, and 90% (95%CI,84–96) and 85.5% (95%CI,76.2–91.3) at 2 years (*p* = 0.4) for 3-fraction and 5-fraction SRS groups, respectively (Fig. 1); for cavities ≥ 3 cm, 1-year and 2-year LC rates were 90% and 87% after 3-fraction SRS and 85% and 83% after 5-fraction SRS (*p* = 0.3), respectively. DBF rates were 45% at 1 year (95%CI, 37.9–54.4%) and 65% at 2 years (95%CI, 51.2–80.4%), with no significant difference between groups. No factors were predictive of LF, although concurrent immunotherapy or targeted therapies was of borderline significance for better LC (*p* = 0.08). On multivariate analysis, stable extracranial disease (*p* = 0.04) and concurrent systemic treatments (*p* = 0.007) were predictors of better DBF.

For the whole population, median OS was 28.3 months, with 1-year and 2-year survival rates of 82.4% (95%CI,77-87.9%) and 61.6% (95%CI,54.2–68.5%), respectively. OS did not differ significantly by groups at 1 and 2 years: 3-fraction SRS, 85% (95%CI,78–92%) and 61.2% (95%CI,52.6–73%); 5-fraction SRS, 79.8% (95%CI,71.6–87.8%) and 60.5% (95%CI,50.3–70.8%). Twenty-nine patients succumbed to their extracranial disease and 81 patients died of progressive intracranial disease. On univariate analysis, stable extracranial disease (*p* = 0.001), breast cancer and melanoma vs. NSCLC histology (*p* = 0.03), KPS (*p* = 0.04), targeted therapies (*p* = 0.02) and immunotherapy (*p* = 0.005) emerged as significant indices of prolonged survival. On multivariate analysis, controlled extracranial disease and concurrent immunotherapy remained independent predictors of survival.

### Risk of symptomatic RN and LMD

Twenty-one (21.8%) patients undergoing 3-fraction SRS and 16 (17%) subjected to 5-fraction SRS group experienced RN (*p* = 0.4), with respective median times to RN of 8 months (range 2–24 months) and 10 months (2–21 months). Diagnosis of RN was confirmed by histology in 14 patients. RN occurred in 16.8% (95%CI,10.2–26.3%) and 23.1% (95%CI,15.4–33.7%) of cavities after 3-fraction SRS, and 12.6% (95%CI,7.3–21.4%) and 17.7% (95%CI,11.2–27.8%) of cavities after 5-fraction SRS (*p* = 0.3), respectively, after 1 and 2 years; for cavities ≥ 3 cm, respective 1-year and 2-year RN rates were 19.5% and 26,4%, and 15.2% and 18,6%, respectively (*p* = 0.3).

**Fig. 2:**
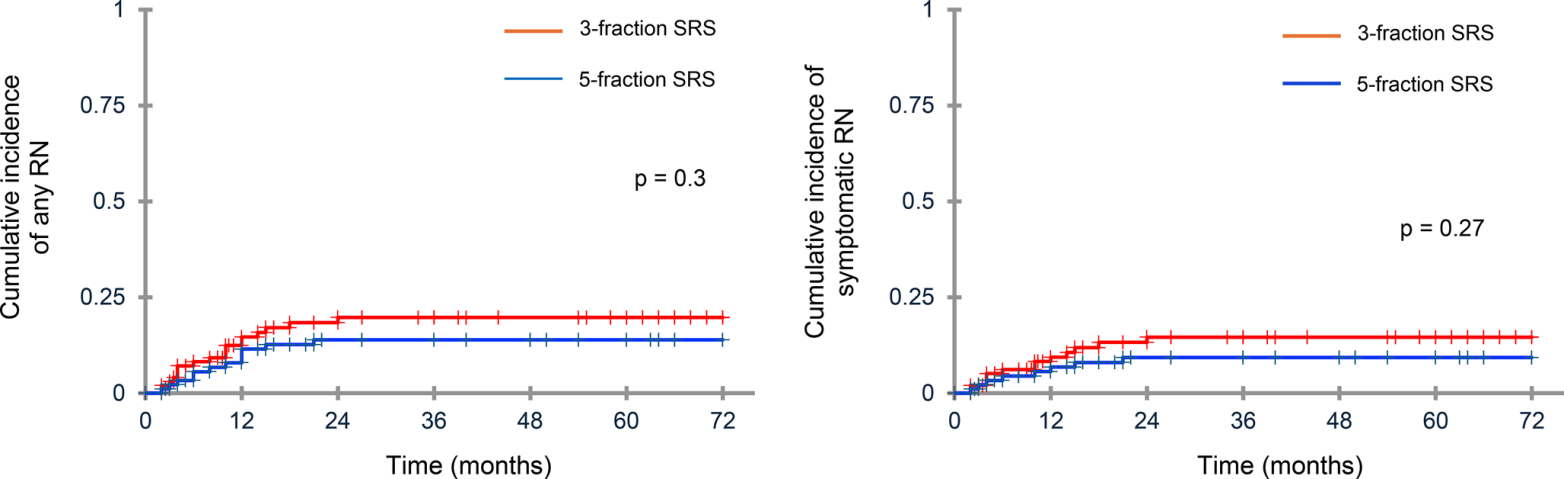
Cumulative incidence of any (**A**) and symptomatic (**B**) radionecrosis after 3-fraction and 5-fraction stereotactic radiosurgery (SRS). The difference between groups was not significant (*p* = 0.3). Censored data are denoted by tick marks

Grade 2 and 3 sRN occurred in 11 (11.4%) and 5 (5.2%) patients after 3-fraction SRS and in 8 (8.5%) and 4 (4.2) patients after 5-fraction SRS, requiring surgery or medical treatment with steroids and bevacizumab. No grade 4 or 5 events occurred. The 1- and 2-year sRN rates were 12.8% (95%CI,8.3–22.4%) and 17.9% (95%CI,12.3–28.9%), respectively, after 3-fraction SRS, and 9.8% (95%CI,6.5–19.1%) and 13.0% (95%CI,7.3–22.2%), respectively, after 5-fraction SRS (*p*= 0.3) (Fig. 2). Respective 1- and 2-year grade 3 sRN rates were 5.1% and 7.6% and 4.4% and 6.5%. Neurological deficits included seizure (*n* = 6), motor deficits (*n* = 11), cognitive deficits (*n* = 12), and speech deficits (*n* = 3).

On univariate analysis, cavity volume, concurrent immunotherapy and ADCs were significantly correlated with the development of symptomatic RN. On multivariate analysis, cavity volume (*p* = 0.055) and concomitant immunotherapy (*p* = 0.07) showed a trend toward the development of sRN.

**Fig. 3:**
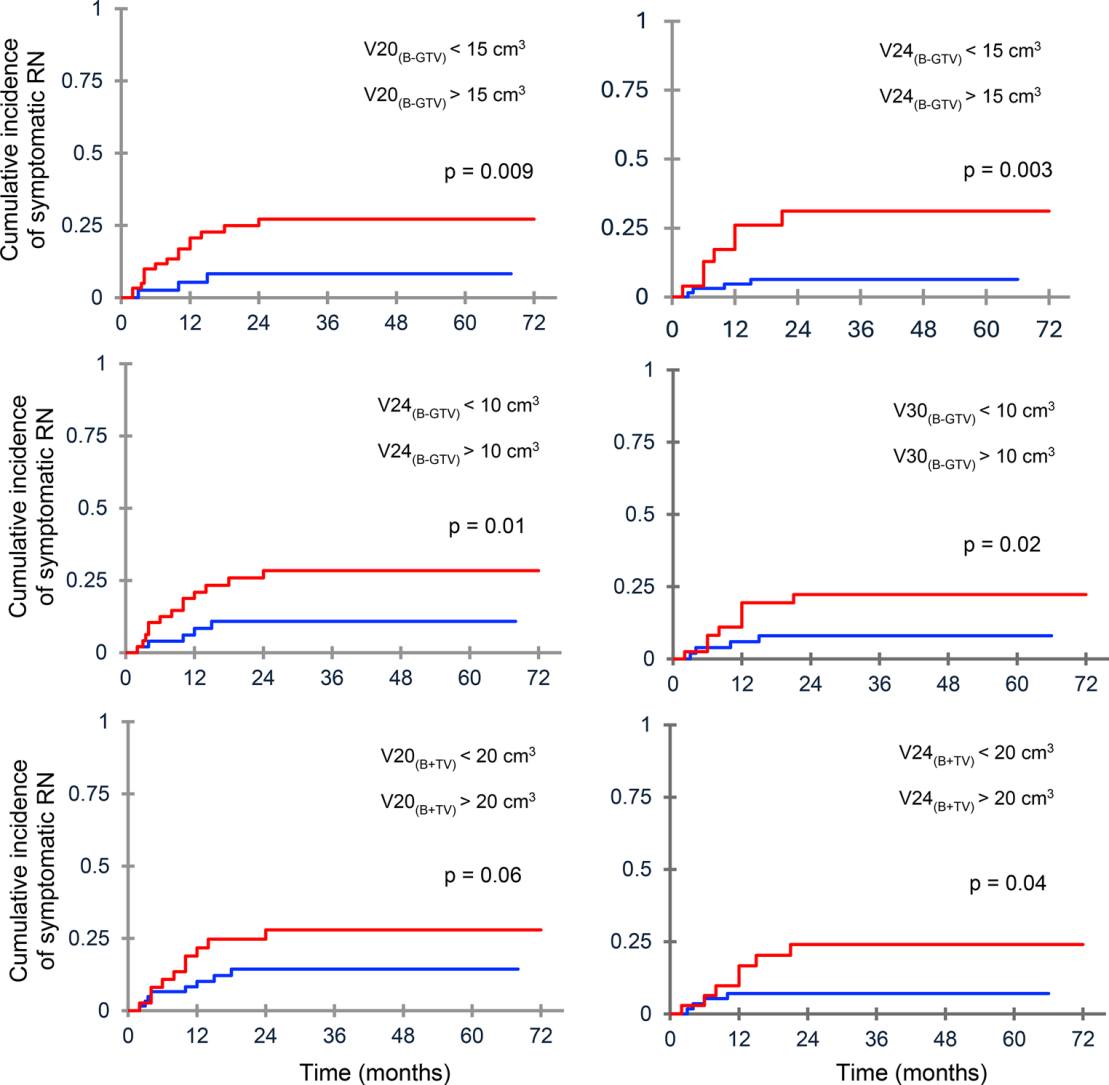
Cumulative incidence of symptomatic radionecrosis (RN) stratified by dose-volume parameters. Following 3-fraction SRS, brain less GTV < 10 cm^3^ receiving < 24 Gy (V24_(B−GTV)_) (**A**) and < 15 cm^3^ receiving < 20 Gy (V20_(B−GTV)_) (**B**) were significantly associated with a lower risk of brain necrosis; in contrast, brain plus target volume receiving 20 cm^2^ (V20_(B+TV_)) (**C**) was not predictive of RN. Following 5-fraction SRS, brain less GTV < 10 cm^3^ receiving < 30 Gy (**C**) and < 15 cm^3^ receiving 24 Gy (V24_(B−GTV)_) (**D**), and brain plus target volume receiving 20 cm^3^ (V24_(B+TV)_) (**F**) were significant predictors of better outcome

Based on previous published risk prediction models, we analyzed the impact of different brain dose-volume constraints on the risk of sRN. In 3-fraction SRS group, V_20(B−GTV)_) < 15 cm^3^, V_24(B−GTV)_, < 10 cm^3^ and V_20(B+TV)_ < 20 cm^3^ were significantly associated with < 10% risk of symptomatic RN (Fig. 3). V_20(B−GTV)_ emerged as the most significant predictor of sRN; the 1-year incidence was 7.1% for V_20(B−GTV)_ ≤ 15 cm^3^ and 22.3% for V_20(B−GTV)_ > 15 cm^3^ (*p* = 0.009), being 19.2% and 30.4% for V_20(B−GTV)_ of 15–30 cm^3^ and > 30 cm^3^, respectively. In 5-fraction SRS group, V_30(B−GTV)_ < 10 cm^3^, V_24(B−GTV)_ < 15 cm^3^, and V_24(B+TV)_ < 20 cm^3^ were associated with significantly lower risk of symptomatic RN (Fig. 3). V_24(B−GTV)_ < 15 cm^3^ and V_30(B−GTV)_ < 10 cm^3^ were most significant constraints; the incidence of symptomatic RN was 4.8% for V_24(B−GTV)_ ≤ 15 cm^3^ and 27.3%% for V_24(B−GTV)_ > 15 cm^3^ (*p* = 0.003). For V_30(B−GTV)_, the incidence of RN was 5.9% for a volume ≤ 10 cm^3^ and 22.3% for a volume > 10 cm^3^ (*p* = 0.02).

LMD occurred in 30 of 190 (15.7%) patients at a median time of 8 months (range, 3–33 months) after surgical resection. Sixteen patients in 3-fraction and 14 patients in 5-fraction SRS group developed LMD. The 1-year LMD cumulative rates were 13.5% (95%CI, 7.3–21%) and 13.8 (95%CI, 8.2–23.3%) in 5-fraction SRS group (*p* = 0.7). Among patients who developed LMD, a nodular pattern occurred in 58% and classical LMD in 42%. The median survival time from the time of diagnosis of LMD was 10 months (range 1–33 months), being 15,6 months for nodular LMD and 5.7 months for classical LMD. On univariate analysis, no factors were associated with the development of LMD; however, location (posterior fossa versus supratentorial location) and proximity to the pial surface (< 1 cm) showed a trend toward increased risk of LMD.

## Discussion

Results of this study, in which fractionated SRS using 27 Gy in three fractions or 30 Gy in 5 fractions was delivered to the surgical bed in patients with resected BMs, indicate that both SRS schedules provide excellent LC with a low risk of sRN. With a median follow-up of 20.5 months, cumulative LC rates were 93% and 90% for the 3-fraction SRS group and 90.5% and 85.5% for the 5-fraction SRS group, respectively, at 1 and 2 years. Our results are consistent with previous studies indicating that 24–27 Gy in 3 fractions or 30–35 Gy in 5 fractions provide high LC for resected BMs [12, 13, 15–19].

A dose-response relationship has been reported for both single-fraction and fractionated SRS using linear-quadratic (LQ) and linear quadratic cubic (LQC) models [27–29]. Based on the LQC model with an α/β ratio of 10, we observed a 1-year LC rate >90% after 3- or 5-fraction SRS, corresponding to a BED10 of 47.25 Gy and 46.05 Gy, respectively. De Boisanger et al. [29] prospectively analyzed post-SRS dose-response results in 91 patients with 471 BM treated with one or three fractions using the LQC model and an α/β ratio of 10. Based on the regression model, 71.5% of those receiving BED10 of 35 Gy were predicted to achieve LC at 9 months. This increased to 90.70% for BED10 of 45 Gy, and 97.4% for BED10 of 55 Gy. in a systematic review of 11 studies including 1079 patients with 1557 BMs, Wiggenraad et al. [27] found that a BED12 >40 Gy was required to achieve 1-year LC rates ≥ 70%, corresponding to 20 Gy in a single fraction or 25.5 Gy in 3 fractions using an α/β ratio of 12. A similar correlation was demonstrated between BED10 and LC by applying the traditional LQ model [30]. in contrast, lower SRS doses, such as 5 × 5 Gy or 3 × 7 Gy corresponding to BED10 < 40 Gy, have been associated with poorer LC [31–34]. Overall, our study confirms that a BED10 >45 Gy, corresponding to doses of 27 Gy in 3 fractions or 30 Gy in 5 fractions, should be recommended to achieve a high LC.

For both groups, LC for resection cavities 2–3 cm in diameter and >3 cm was similar. The LC rates of 90% after 3-fraction SRS and 85% after 5-fraction SRS to the surgical beds >3 cm compare favorably with those reported after single-fraction SRS. In a recent randomized study of postoperative SRS versus observation in 128 patients undergoing *en bloc* gross total resection for 1 to 3 BMs between 2009 and 2016 at the University of Texas M.D. Anderson Cancer Center, Majan et al. [8] observed 12-month tumor-free local recurrence rates of 91% for cavities with a maximum diameter of ≤ 2.5 cm treated with 16 Gy, 40% for cavities 2.5 to 3.5 cm treated with 14 Gy, and 46% for those with tumors >3.5 cm treated with 12 Gy (*p* = 0.0002). In another randomized study comparing SRS with WBRT in 194 patients with resected BMs, Brown et al. [8] reported worse LC for postoperative cavity volumes >20 ml subjected to radiation doses < 15 Gy. The excellent LC reported in our study supports the use of hypofractionated SRS, either 3 or 5 fractions, for resection cavities >3 cm in diameter.

In a recent review of 69 articles reporting clinical outcomes following postoperative single-fraction and fractionated SRS in adult patients, an estimated variable risk of sRN at 1 year ranging from 2% to 25% was reported [12]. In our study, the development of grade 2 and 3 RN, diagnosed by imaging or histology, occurred in 11.3% and 15.2% of patients, respectively, after 1 and 2 years, with no significant differences between groups. The estimated risk of sRN is consistent with those observed in previous series using 27 Gy in 3 fractions or 30 Gy in 5 fractions (17–19,35) and indicates that hypofractionated schedules can be safely used for large cavity volumes, even in combination with systemic treatments.

An estimated 1-year risk of sRN < 8% was observed for V_20(B−GTV)_ < 15 cm^3^ and V_20(B−GTV)_ < 10 cm^3^ (3-fraction group) and V_24(B−GTV)_ < 15 cm^3^ and V_30(B−GTV)_ < 10 cm^3^ (5-fraction group). Using V_30(B−GTV)_ as predictive factor for sRN in 118 surgical cavities treated with a median of 30 Gy in 5 fractions, Faruqi et al. [18] showed a risk of 13% for volumes < 10.5 cm^3^ and 61% for volumes ≥ 10.5 cm^3^ at 1 year. With the same regimen, Andruska et al. [35] observed a risk of sRN of 21% for V_30(B−GTV)_ >10 ml and 2% for V30 ≤ 10 cm^3^ at 2 years in 83 patients with 117 intact BMs (*p* = 0.007). Other studies found a significant correlation between brain plus target volume and sRN [19, 26]. Upadhyay et al. [19] showed that intact and resected BMs receiving V_20(B+TV)_ < 20 cm^3^ and V_23(B+TV)_ < 15 cm^3^ were associated with < 10% any grade RN and < 5% of grade 3 RN, respectively. In a pooled analysis of 888 patients with 982 lesions treated with 3-fractions SRS, Milano et al. [26] reported that V_20(B+TV_ (3 fractions) < 20 cm^3^ or V_24(B+TV)_ (5 fractions) < 20 cm^3^ was associated with < 10% risk of any RN and < 4% risk of sRN. In our study, V20-24_(B+TV)_ were less predictive than brain minus GTV receiving 20–24 Gy. Slight differences in the impact of different dose-volume constraints may be due at least in part to the inclusion of only resected BMs in our series, the use of concurrent systemic treatment in most patients, and the application of different GTV to CTV/PTV margins. Overall, brain dose-volume constraints, including V20, V24, and V30, are useful for predicting the risk of symptomatic radiation necrosis, and their impact should be evaluated in large prospective studies and implemented in clinical practice.

LMD occurred in 30 (15.7%) patients, with no differences between groups. This is consistent with those seen in series reporting an incidence of 7% to 28% after postoperative SRS of resected BMs [7, 8, 11, 36–41]. Our results confirm the relatively high incidence of LMD after surgical resection and SRS, which is often seen as focal nodules adherent to the dura or pia near the irradiated cavity [42–44]. Recognizing this pattern of meningeal spread is of importance because it may be associated with better outcome compared with classical LMD [42, 44].

In conclusion, hypofractionated SRS to the resection cavity using 3 × 9 Gy and 5 × 6 Gy is associated with high LC and low sRN risk. V_20(B−GTV)_ < 15 cm^3^ and V_20(B−GTV)_ < 10 cm^3^ for 3-fraction SRS, and V_24(B−GTV)_ < 15 cm^3^ and V_30(B−GTV)_ < 10 cm^3^ for 5-fraction SRS should be validated in large prospective studies and implemented in clinical practice to reduce the risk of sRN.

## Data Availability

Data generated or analyzed during this study are available from the corresponding author upon reasonable request.
